# Betulinic Acid for Glioblastoma Treatment: Reality, Challenges and Perspectives

**DOI:** 10.3390/ijms25042108

**Published:** 2024-02-09

**Authors:** Sílvia Fernandes, Mariana Vieira, Cristina Prudêncio, Ricardo Ferraz

**Affiliations:** 1 Center for Translational Health and Medical Biotechnology Research (TBIO), School of Health (ESS), Polytechnic University of Porto, Rua Dr. António Bernardino de Almeida, 400, 4200-072 Porto, Portugal; smf@ess.ipp.pt (S.F.); cprudencio@ess.ipp.pt (C.P.); 2 Center for Research on Health and Environment (CISA), School of Health (ESS), Polytechnic University of Porto, Rua Dr. António Bernardino de Almeida, 400, 4200-072 Porto, Portugal; 3 Ciências Químicas e das Biomoléculas, School of Health (ESS), Polytechnic University of Porto, Rua Dr. António Bernardino de Almeida, 400, 4200-072 Porto, Portugal; 4 LAQV-REQUIMTE, Departamento de Química e Bioquímica, Faculdade de Ciências, Universidade do Porto, Rua do Campo Alegre, 687, 4169-007 Porto, Portugal

**Keywords:** betulinic acid, glioblastoma, apoptosis, invasiveness, blood–brain barrier, drug delivery

## Abstract

Betulinic acid is a naturally occurring compound that can be obtained through methanolic or ethanolic extraction from plant sources, as well as through chemical synthesis or microbial biotransformation. Betulinic acid has been investigated for its potential therapeutic properties, and exhibits anti-inflammatory, antiviral, antimalarial, and antioxidant activities. Notably, its ability to cross the blood–brain barrier addresses a significant challenge in treating neurological pathologies. This review aims to compile information about the impact of betulinic acid as an antitumor agent, particularly in the context of glioblastoma. Importantly, betulinic acid demonstrates selective antitumor activity against glioblastoma cells by inhibiting proliferation and inducing apoptosis, consistent with observations in other cancer types. Compelling evidence published highlights the acid’s therapeutic action in suppressing the Akt/NFκB-p65 signaling cascade and enhancing the cytotoxic effects of the chemotherapeutic agent temozolomide. Interesting findings with betulinic acid also suggest a focus on researching the reduction of glioblastoma’s invasiveness and aggressiveness profile. This involves modulation of extracellular matrix components, remodeling of the cytoskeleton, and secretion of proteolytic proteins. Drawing from a comprehensive review, we conclude that betulinic acid formulations as nanoparticles and/or ionic liquids are promising drug delivery approaches with the potential for translation into clinical applications for the treatment and management of glioblastoma.

## 1. Medicine and Plant-Derived Compounds

Medicinal plants or herbs have been employed in medicine since ancient times and across different cultures [1], and the interest in plant-derived compounds in the context of their potential use in medicine is currently growing. Beyond their therapeutic applications, these herbs have found use in various areas, including the areas of nutrition, flavorings, beverages, dyeing, repellents, perfumes, cosmetics, charms, smoking, and other industrial applications [1]. The therapeutic efficacy of these plants is essentially attributed to the bioactive compounds present in their chemical composition [2,3]. These chemical entities, whether singular or in combination, are extracted and utilized for healing or preventing various human pathologies [2,4,5]. For instance, Lamiaceae plants are cultivated globally, primarily for medicinal and culinary purposes [6], and extensively studied as natural sources of antioxidants due to their rich polyphenol content. Their potent bioactivity and relatively low toxicity make them valuable ingredients in complementary medicine and nutritional supplements [6]. Another important example is *Artemisia annua* L., the plant responsible for producing artemisinin, a vital treatment for malaria, earning the 2015 Nobel Prize in Physiology or Medicine for its discovery [7]. These examples underscore the continued dominance of plants in contemporary therapeutic approaches [2,4]. While plants remain paramount in natural product applications for medicine, alternative sources include microorganisms, as demonstrated by Fleming in 1929 [8], marine products like spongouridine and spongothymidine isolated from a Caribbean sponge in the early to mid-1950s [9], and even animals [10].

One significant challenge with natural products is that they are produced to meet the needs of the organisms producing them, rather than the requirements of medicinal practice. This makes it difficult to enhance potency, selectivity, and pharmacokinetics [9,11]. Additionally, natural products often exhibit low solubility in water, prompting the development of new formulations and drug delivery methodologies to overcome this limitation, as better explored and described below.

## 2. Betulin and Betulinic Acid

**Figure 1:**
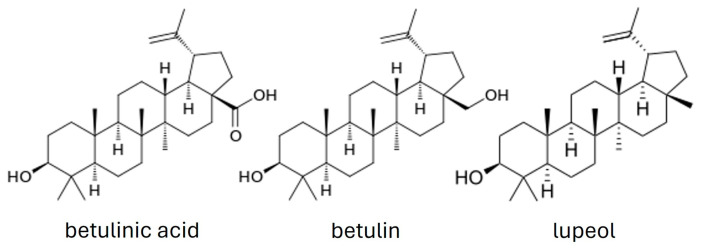
Chemical structures of the naturally pentacyclic lupine-type triterpenoid.

Betulin is a compound of great interest since it can be utilized as a precursor for the synthesis of novel derivatives with improved pharmacological properties and potential use in medicine. Betulinic acid (BA, 3-beta-hydroxy-lup20(29)-en-28-oic acid) is a plant-derived pentacyclic lupine-type triterpenoid that can be obtained through extraction from birch trees, chemical synthesis, or microbial biotransformation [12]. The chemical structures of BA, betulin, and lupeol are schematically represented in Figure 1.

BA can be found in Menyanthes trifoliata, a swampy plant [13], and it is obtained through methanolic [14] or ethanolic extraction [15] from different plant sources [13], although the extraction process from plants is difficult. The main problems are related to the quantity of BA available in the plant. The birch bark only has between 0.002% and 2% BA, and the yield is low; also, it is not a sustainable and environmentally friendly process [12].

Other common triterpenoid acids, such as oleanolic and ursolic acid, show weak anti-inflammatory and antitumor properties [9,16]. In the case of BA and its derivatives (betulin and 23-hydroxybetulinic acid), they are characterized by the presence of four rings and by one of five carbons bearing an isoprenyl group, and they belong to the lupan group. BA has been extensively studied for its potential therapeutic properties, which include anti-inflammatory properties, anti-viral activity against certain viruses, including HIV and influenza viruses, hepatoprotective properties, as well as antimalarial and antioxidant activity [17,18]. As far as we know, the total synthesis of BA has not been described yet. Although lupeol, a very similar lupane, already has been synthesized [19,20].

The production of BA for commercial purposes is through semisynthesis, via the oxidation of the primary hydroxyl group of betulin [21]. Betulin has the advantage of being more abundant in birch trees (up to 34%) [21,22] and the disadvantage of having less biological activity [23] when compared to BA. The most common synthesis process of BA from betulin is via Jones oxidation of the two hydroxyl groups, followed by the reduction of the ketone by sodium borohydride [12,23,24]. 

In order to prevent isomerization, the five-step approach could be employed. Initially, the primary alcohol is protected, followed by acetylation of the secondary alcohol. Subsequently, deprotection and oxidation to the carboxylic acid take place, followed by deacetylation to produce BA [12,23,24].

Another strategy for producing BA involves employing biotechnological biosynthesis techniques [12,13]. First, the lupeol synthesis through 2,3-oxidosqualene cyclization occurs, followed by the oxidation in the primary hydroxyl catalyzed by the enzymes of cytochrome P450 (CYP) [13,25]. With the discovery of the genes encoding the enzymes critical for these reactions, more possibilities are being found to produce BA [12,15].

### Biological Properties of Betulinic Acid

Several important properties have been described in the literature for BA, such as anti-inflammatory, anti-viral, anti-malarial, immunomodulatory, anti-fibrotic, and antitumor properties [17,18]. In addition, an important feature of the BA is that it can cross the blood–brain barrier (BBB), overcoming one of the main barriers to the treatment of neurological pathologies and/or disorders. Regarding central nervous system (CNS) disorders, some molecular insights have been found with BA treatment as well as important neuroprotective properties. In cerebral ischemia stroke model rats, for example, pretreatment with BA not only lessens cerebral injury by lowering oxidative stress, but also stimulates the SIRT1/FoxO1 pathway to repress autophagy and ameliorates cerebral injury [26]. Another example is that BA can greatly increase functional recovery after a spinal cord injury in mice by inhibiting pyroptosis, an inflammatory form of programmed cell death [27]. BA can also dramatically reduce catalepsy and stride length, while increasing the brain’s dopamine content, glutathione activity, and catalase activity in hemiparkinsonian rats [28], and, in Alzheimer’s disease rat models, it can improve neurobehavioral impairments [29].

BA has exhibited great neuroprotective properties. It is able to attenuate lipopolysaccharide-induced neuroinflammation by promoting M2 anti-inflammatory polarization of the microglia [30], and diminish the redox imbalance and cholinergic and proteolytic enzyme activities in iron-mediated neurotoxicity [31]. After neuroinflammation induced by the mycotoxin T-2, BA improves cognitive ability and neurotransmitter levels, and protects from brain damage by lowering reactive oxygen species (ROS) levels, enhancing brain tissue’s antioxidant capacity, and preventing the release of inflammatory cytokines [32].

Furthermore, BA has exhibited considerable potential as an anticancer agent. The agent was initially known for its high cytotoxicity against human melanoma cancer cells, but later studies also suggest BA exhibiting extraordinary effects on a variety of malignancies, including bladder [33], breast [34,35], ovarian [36,37], gallbladder [38], and colorectal [39] and gastric cancers [40], with IC50 values between 1 and 13.0 µg/mL. These effects have also been shown to be selective against tumor cells, although the specific mechanisms behind this cellular selectivity are still unknown [12,13].

BA can decrease the mitochondrial outer membrane potential (MOMP), increase the production of ROS, and inhibit antiapoptotic proteins while increasing the level of proapoptotic ones, thereby inducing cell apoptosis [41]. The compound can inhibit the signal transducer and activator of transcription (STAT) 3 signaling pathways, involved in differentiation, proliferation, apoptosis, metastasis formation, angiogenesis, and metabolism, and the NF-kB signaling pathway, a transcription factor commonly overexpressed in tumors that regulates processes such as cell survival, deoxyribonucleic acid (DNA) transcription, and cytokine production [41,42]. Additionally, BA has shown an ability to control cancer growth through the modulation of Sp transcription factors, inhibit DNA topoisomerase [2,10], induce autophagy [42], and inhibit the epithelial-to-mesenchymal transition (EMT) [41,43]. BA has also been associated with an antiangiogenic response under hypoxia conditions, through the STAT3/hypoxia-inducible factor (HIF)-1α/vascular endothelial growth factor (VEGF) signaling pathway [44].

Regarding glioblastoma, BA effects have already been the subject of some research. Besides its known effect when used alone, BA has shown great potential as an adjuvant to therapy since its use combined with standard treatment of chemotherapy and irradiation can enhance their cytotoxic effect on cancer cells [13,41,45,46]. These aspects will be further detailed below.

## 3. Glioblastoma

Glioblastoma is a malignant subtype of gliomas that are common brain tumors arising from glial cells. It accounts for 14.5% of all primary brain tumors and 48.5% of malignant primary brain tumors, and has an incidence of 3.2 to 4.2 per 100,000 people [47]. This challenging and aggressive form of cancer develops from astrocytes and is categorized as a grade IV tumor, the most aggressive classification by World Health Organization (WHO) standards [48]. The standardized treatment plan in place involves surgery for total resection, followed by a combination of radiation therapy and chemotherapy with Temozolomide (TMZ) as the first-line agent [49]. Despite this aggressive and multi-approach treatment, the average patient has a survival length of only 15 months, with the five-year survival rate for this neoplasm being approximately 5% [47,50,51,52,53]. Few specific risk factors have been linked to glioblastoma; however, it is known that exposure to ionizing radiation is one of them, while a history of atopic diseases can be related to a reduced risk [47,54,55].

There are a number of genomic alterations associated with glioblastoma, the most commonly described being isocitrate dehydrogenase (*IDH*), fibroblast growth factor receptor (*FGFR*), a-thalassemia/mental-retardation-syndrome-X-linked (*ATRX*), and tumor protein P53 (*TP53*) gene mutations, epidermal growth factor receptor (*EGFR*) gene amplification, loss of heterozygosity of the phosphatase and tensin homolog (*PTEN*) gene and telomerase reverse transcriptase (*TERT*) promoter mutation [47,49,54,55]. These frequent alterations play a crucial role in determining the patient’s outcome. In fact, several of these molecular biomarkers can help establish a prognosis, but the most accepted and used ones include the previously mentioned *IDH* mutations and O(6)-methylguanine-DNA methyltransferase (*MGMT*) promoter methylation status. IDH mutations are associated with better prognosis and higher survival rates, while the *MGMT* promoter plays a role in the response to chemotherapy, as hypermethylation of this promoter decreases the expression of MGMT, a protein that hinders the action of alkylating chemotherapy agents such as TMZ and lomustine [56].

Glioblastoma is also highly prone to recurrence, with a median recurrence time of 6.2 months [57], and in these cases, there is no standard care plan, but only a few options exist that include further surgical resection, systemic therapies such as TMZ rechallenge, lomustine or bevacizumab re-irradiation, and clinical trials [54,55,56].

Many factors complicate glioblastoma’s treatment, as is the case of intertumor and intratumor heterogeneity, as well as its immunosuppressive microenvironment, making it difficult to develop a more directed therapy approach [49,52,53,58].

Additionally, another major obstacle to glioblastoma chemotherapy is the difficulty that therapeutic agents have in successfully crossing the BBB [59]. The selective permeability of BBB is attributed to the presence of tight junctions and active transport mechanisms [60], and even though this barrier is compromised in glioblastoma patients, with the downregulation of tight junction proteins causing it to become leakier [60,61], many therapeutic drugs still cannot cross this barrier, and others cannot reach a concentration that has a therapeutic effect, reducing even further the available drug options for treatment [62,63].

Glioblastoma can develop resistance to therapeutic agents, through mechanisms including the demethylation of the MGMT promoter and the mismatch repair (MMR) deficiency, which too, leads to strong resistance to alkylating agents [60,64]. The presence of glioblastoma stem cells (GSCs) increases resistance to chemotherapeutics as well, mainly due to their overexpression of ATP-binding cassette (ABC) transporters. Their enhanced DNA repair abilities, the overexpression of anti-apoptotic proteins, and the transfer of mitochondria from mesenchymal stem cells (MSCs) to GSCs also contribute to their chemoresistant phenotype [51,65,66,67]. The tumor microenvironment (TME) also plays a role in this resistance by promoting a hypoxic and acidic environment. Hypoxia favors the development and maintenance of CSCs, promoting their chemoresistant phenotype, and stimulating various oncogenic pathways [51,58,68]. Herein, both the endothelial and immune cells that compose the TME and the ECM can also influence and support CSC phenotypes and chemoresistance [69,70].

When considered the tumor’s poor prognosis, therapeutic difficulties, propensity to recurrence, and acquired chemoresistance discussed above emphasize the need to design and develop new or adjuvant therapies for glioblastoma.

### Glioblastoma’s Invasive Profile

Although glioblastoma does not normally metastasize to organs outside the brain, it still invades the local healthy brain tissue along previously established structures such as blood vessels and between neurons and glia [71,72,73].

Some of the biological processes that allow glioblastoma cells to separate from the surrounding tumor tissue and spread to other areas include the adjustment of cells’ adhesion to one another and to the extracellular matrix, cytoskeletal remodeling, communication with host cells, and the EMT [74]. The EMT transition plays an essential role in invasion and is regulated by specific transcription factors, such as Snail, Slug, zinc-finger E-box-binding homeobox (ZEB)1/2, and Twist1/2, that suppress the expression of epithelial markers such as E-cadherin, claudins, occludins, and cytokeratins while increasing the expression of mesenchymal markers such as N-cadherin, vimentin, and fibronectin [72].

The detachment of cells is modulated by adhesion molecules like cadherin, neuronal cell adhesion molecule (NCAM), and integrins. In more aggressive forms of glioma, like glioblastoma, NCAM expression is diminished. These proteins alter the ECM and the production of matrix metalloproteinases (MMPs), which break down cadherins [71]. Cadherins, on the other hand, are involved in cell adhesion processes and their instability, and subsequent loss of cell-to-cell adhesion, facilitating glioma cell mobility [74]. Integrins, whose expression is upregulated in glioblastoma, contribute to the tumor’s invasiveness by facilitating cell movement, acting as receptors, and enabling cells to adhere to stromal cell proteins or those present in the ECM [75].

Moreover, glioblastoma cells can mold the surrounding environment through the secretion of proteolytic molecules like MMPs, A disintegrin and metalloproteinases (ADAMs), urokinase-type plasminogen activator (uPA), and cathepsins, which break down the ECM components and allow the tumor cells to invade [75]. They can also recruit surrounding cells like glioma-associated macrophages (GAMs) and tumor-associated astrocytes (TAAs), which in turn secrete proteases, cytokines, and other pro-invasive factors that stimulate the tumor cells to release MMPs and promote their invasion [76].

Understanding the cellular and molecular processes behind the glioblastoma invasive phenotype will help in the development of novel treatment approaches to lessen the aggressiveness of this type of tumor, which is one of the key features linked to therapy failure.

For instance, a study investigating the role of LDHA and LDHB subunits of lactate dehydrogenase (LDH) and lactate metabolism in glioblastoma tumor growth and invasion has shown that lactate is an important metabolic factor in tumor growth and invasiveness, and that Stiripentol, an antiepileptic drug that inhibits LDHA and LDHB activity, efficiently reduces glioblastoma’s development and invasion [77], demonstrating potential as a new therapeutic or adjuvant agent. Another example is eriodictyol, a natural flavonoid that has also demonstrated an ability to suppress migration and invasion by downregulating the P38 MAPK/GSK-3β/ZEB1 signaling pathway [78]. Besides, chrysomycin A, an antibiotic from Streptomyces is reported to have antitumor and anti-tuberculous activities, and its mechanism of action was investigated in a study conducted with glioblastoma U251 and U87-MG human cells. Treatment with chrysomycin-A significantly inhibited the growth of glioblastoma cells and weakened the ability of cell migration and invasion by downregulating the expression of slug, MMP-2, and MMP-9. Furthermore, downregulation of Akt, p-Akt, GSK-3β, p-GSK-3β, and their downstream proteins β-catenin and c-Myc, was also observed in human glioblastoma cells. In conclusion, chrysomycin-A may inhibit the proliferation, migration, and invasion of glioblastoma cells through the Akt/GSK-3β/β-catenin signaling pathway [79].

## 4. Betulinic Acid and Glioblastoma

### 4.1. Betulinic Acid Effects

BA effects on glioblastoma cells have already been the subject of some research and some interesting insights have been found for the compound as an antitumor agent, impacting important cellular signaling cascades such as proliferation, migration, and apoptosis. Conducted research revealed that BA improved the effects of both chemotherapy and radiotherapy, increasing the sensitivity of cancer cells to chemotherapy or irradiation agents [41,46,80,81]. In this sense, BA can selectively target tumor cells, and enhance the cytotoxic effect of TMZ when used in combination, including in TMZ-resistant cell lines [46]. Other important drugs extensively used in brain cancer chemotherapy are platinum-based drugs. Regarding nervous system tumors, the challenge in using these drugs lies in their dose-dependent toxicity. The concurrent administration of cisplatin with BA and its derivatives in human malignant glioma cells resulted in a decreased cell survival rate, both under normal oxygen and hypoxic conditions [41]. Few studies have examined the combination of BA or its derivatives using radiotherapy in glioblastoma [80], but it is suggested that BA could be a promising drug for increasing radiosensitization of cells, as already observed, for instance, in oral squamous cell carcinoma [41].

**Figure 2:**
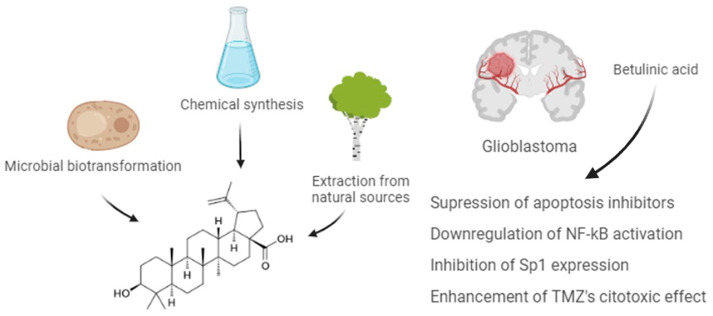
Betulinic acid (BA) is a plant-derived pentacyclic lupine-type triterpenoid that can be obtained through chemical synthesis or microbial biotransformation from birch trees. Due to the ability to cross the blood–brain barrier, BA is a potent therapeutic tool to be used in the clinical management of glioblastoma due to the selective cytotoxicity, anti-fibrotic, and other antitumoral effects well documented in the literature. *Image created with BioRender.com*.

BA exhibits significant in vitro cytotoxicity in a variety of tumor cell lines but also inhibits the growth of solid tumors in vivo. The exact molecular mechanism is still unclear, but BA can inhibit proliferation and induce apoptosis in glioblastoma cells by downregulating NF-κB activation and suppressing apoptosis inhibitors [82,83], and inhibit Sp1 expression, a pro-survival transcription factor [84]. Overall, the evidence suggests a great potential for BA as a therapeutic agent for glioblastoma, according to the illustration in Figure 2. As observed for other tumor cell lines, including breast cancer cell lines, the effect of BA on the expression of proteolytic enzymes, such as MMP-2 and MMP-9, as well as cell adhesion molecules involved in migration should be further evaluated to identify and explore possible targets for BA treatment of glioblastoma [85].

### 4.2. Betulinic Acid Derivative Effects

Modifications at positions C-3, C-20, and C-28 are crucial for BA bioactivity and cytotoxicity as an anticancer agent, and can also increase its solubility without affecting pharmacological activity [41,44,86,87]. Recently, several semi-synthetic derivatives of BA have been generated, showing promising activity against glioma cells, as is the case of BA10 and NVX-207. B10, a BA ester derivative coupled with D-glucose, has been shown to be more effective than BA and induced apoptosis in different tumor cell lines. The compound was used to explore the molecular mechanism underlying the anticancer effect of B10 in glioma cells, and causes a significant reduction in the implanted tumor weight and volume in mice after 25–50 mg/kg administration. Specifically, B10 activated apoptosis through induction of mitochondrial dysfunction and pointed SIRT1-FOXO3a-Bim/p53 upregulated modulator of apoptosis (PUMA) axis as a novel therapeutic target for glioma treatment [88].

Another study assessed the effectiveness of another BA derivative, NVX-207, a betulinic ester, when compared to BA. Results showed that both derivatives have higher cytotoxicity in glioma cell lines under normoxic conditions. Moreover, NVX-207 displayed the strongest effect regardless of the oxygen conditions [45]. Other studies have shown a strong cytotoxicity both in vitro and in vivo for NVX-207 derivative. The mechanisms involved suggested that NVX-207 has modulatory effects in lipid metabolism, with an upregulation of genes coding for insulin-induced gene 1 (*Insig-1*), low-density lipoprotein receptor (*LDL-R*) and 3-hydroxy-3-methylglutaryl coenzyme A (*HMG-CoA*) [89]. Another important finding was that NVX-207 and B10 treatments resulted in reduced rates of glioblastoma cell migration, with the strongest inhibition effect on migration for NVX-207 [45].

Lysosomal cell death pathway activation seems to be important for BA10 cytotoxicity [80]. The study conducted by Fisher et al. investigated the influence of hypoxia, nutrient deprivation, and current standard TMZ and irradiation therapies on B10 cytotoxicity. The human glioma cell lines used were exposed to B10 alone or in combination with different treatment conditions and showed enhanced cytotoxicity under hypoxia. B10 treatment results in a shift of the cathepsin Z and cathepsin B (CTSB) enzymes from lysosomes to cytoplasm and nucleus, further corroborating that B10 induces lysosomal permeabilization. Considering the significant role of hypoxia in therapy resistance and malignant progression mentioned above, B10 emerges as a potentially promising approach for hypoxic tumors, such as malignant glioma [80]. Other derivatives have been produced and evaluated, with great in vitro results when compared to standard chemotherapeutic agents and irradiation [90]. However, studies are still in the exploratory stage because of a significant limitation in applying it therapeutically.

### 4.3. Delivery Strategies for Betulinic Acid Application

Despite having great potential as a therapeutic agent, it is hard for BA to fulfill the requirements for adequate water solubility, maintaining both significant cytotoxicity and selectivity for tumor cells. Consequently, endeavors have been made to enhance the solubility of existing molecules and explore new and more efficient modes of administration, such as liposomes, nanoconstructs, and ionic liquids, including for brain tissue access [16,91,92].

Based on its extraordinary potential as an antitumor agent, BA was involved in phase I/II clinical trials to assess its effectiveness and safety, as is the case of melanoma lesions topical treatment [44]. However, in what concerns BA as an anti-glioblastoma agent, it has not been widely used in the treatment of clinical tumor diseases, due to its reduced water solubility and bioavailability [41,93].

Intravenous injection represents one of the least invasive drug delivery routes to the brain, but its effectiveness has been significantly compromised by the existence of the BBB. Indeed, drug delivery to glioblastoma is notoriously difficult due to the inability of most drugs to traverse this tight barrier and penetrate the tumor tissue. Drawing inspiration from the increased ability of some agents to cross the BBB, this problem could be overcome through the development of new delivery strategies, like nanoparticle formulations or ionic liquid derivatives of BA [16,84].

Regarding BA ionic liquid formulations, the scientific works recently conducted on this topic suggest that those formulations can potentiate the therapeutic action of available antitumor drugs, which makes them valuable tools for fighting cancer. For instance, the solubility of a choline betulinate was found to be at least 100 times greater in water than free BA. In vitro studies using different cell lines have shown that organic salts synthesized displayed greater antiproliferative activity when compared to the parent BA [16]. Moreover, other study investigated the effects of BA ionic derivatives against several cancer cell lines, such as epidermoid carcinoma, breast adenocarcinoma, melanoma, and neuroblastoma. This study revealed new ionic derivatives of BA exhibited high cytotoxicity levels toward several cancer cell lines based on the most common viability and cytotoxicity assay methods, when compared to BA. The authors ascribed the enhanced outcomes to the preservation of the BA structure, coupled with the attainment of increased water solubility [94]. Similar results were obtained using a different approach [94]. In this work, the novel 3-indolyl substituted betulin derivatives were produced and assessed for antitumor activity against several human cancer cell lines (melanoma, breast cancer, colorectal adenocarcinoma, lung cancer) and overall, increased cytotoxicity levels were registered when compared to the parent BA. Although proposed for a different type of malignant cells, the results of this in vitro study show that the indole-functionalized triterpene EB367 could be regarded as a promising candidate for further analysis as well as used as a skeleton structure for developing new chemotherapeutics against glioblastoma [95].

Currently, for their stability and safety, BA nanoparticles are one of the most promising approaches with the potential to be translated into clinical use to improve the management of glioblastoma [84,96]. Nanotechnology-based drug delivery systems have undergone extensive evolution and research in recent years, and we can find studies conducted with nanoparticles composed of gold, carbon, lipid, and hybrid nanoparticles, among several other compositions [41,81,93,97,98]. The most promising nanoparticles identified thus far are hybrid polymer nanoformulations with ligands targeting receptors present in glioma cells. These formulations exhibit biochemical versatility by combining various properties of different nanomaterials in a single nanoformulation [93,97].

BA nanoparticles have already been proven since they could effectively cross the BBB and can be used for therapeutic use. Indeed, different nanoparticle properties influence their capacity for crossing the BBB, their ability to encapsulate different therapeutic agents, and their serum half-life [93]. Studies conducted show BA nanoparticles efficiently cross the BBB and penetrate the mice’s brains, significantly reducing ischemia-induced infarction as an antioxidant agent [96]. Regarding glioblastoma treatment, BA nanoparticles were injected in an intracranial xenograft model and showed to have important antitumoral effects [84]. Based on the research using this model, the therapeutic action of BA is mainly related to suppression of the Akt/NFκB-p65 signaling, mediated by CB1/CB2 cannabinoid receptors [84]. Another study explored a technology approach of a synthetic protein nanoparticle based on polymerized human serum albumin. In combination with ionized radiation, nanoparticles carrying siRNA against signal transducer and activation of transcription 3 factor result in tumor regression and long-term survival in 87.5% of glioblastoma mice [99].

Indeed, the application of nanoparticles for the delivery of siRNA is a promising strategy. This enables the downregulation of the expression of target genes with protumor activity. Additionally, it facilitates the use of other immune-mediating particles that induce a transformation in the inherently immunosuppressed tumor microenvironment, making it more susceptible to targeting by the immune system [81,93]. Functionalization of nanoparticles with targeted receptors and/or proteins to counteract glioblastoma malignant profile have been demonstrated, but few (less than 10%) have reached phase I/II clinical trials and almost none of them have progressed to phase III [81,100]. Those studies have often stalled at the animal model testing stage and the main causes are the current lack of evidence concerning the safety of these drugs, potential mid- to long-term toxicity, immunogenicity, and pharmacokinetic and pharmacodynamic profiles. Moreover, the diversity of nanoconstructs used, varying in size, shape, surface charge, and components, renders these nanomaterials highly variable which impacts negatively the standardization of the studies [41,81,93,97].

Overall, the transformation of any therapeutic agent, such as BA, which exhibits limited solubility in aqueous environments, into formulations with increased bioactivity stands as a significant stride toward the creation of orally bioavailable drugs. When considered collectively, these discoveries mark a crucial milestone in the prospective design and development of novel anticancer agents, leveraging the cost-effective production of organic salts and ionic liquids, as well as nanoformulations derived from antitumor compounds.

## 5. Main Conclusions and Future Directions

Glioblastoma is a common malignancy in clinical practice management, and although great progress in medical technology has been made in recent years, the prognosis is still far from the ideal. Thus, the present comprehensive review compiles scientific research about the anti-glioblastoma effects of BA. In brief, BA has an impact on apoptosis inhibitor suppression, downregulation of NF-κB activation, and inhibition of Sp1 expression. Taken together, in what concerns the invasiveness profile prevention, evidence of BA effects suggests that the expression of proteolytic enzymes and cell adhesion molecules involved in migration/invasion tumoral processes should be further explored. Although requiring further investigation, the cellular selectivity shown and the fact that the compound can easily cross the BBB are important properties of BA, offer a prospect for enhancing the clinical management of glioblastoma. Nevertheless, we compile some recent technologies allowing for improved local drug delivery, including for BA and their derivatives. Given their notable antitumoral efficacy and excellent safety profile, there is promising potential for the translation of both nanoparticles and ionic liquid formulations of BA therapeutic applications, and as research in therapeutic delivery systems progresses with promising results, there will be a naturally growing interest and investment which will allow the advancement from preclinical to clinical trials.
